# Liver Resection for Intrahepatic Stones

**DOI:** 10.1155/1990/59151

**Published:** 1990

**Authors:** Roland Andersson, Karl-Göran Tranberg, Stig Beng-Mark

**Affiliations:** Department of Surgery Lund University Lund S-221 85 Sweden

## Abstract

Intrahepatic stones are difficult to manage, especially when they are associated with bile duct stricture, cholangitis and destruction of liver parenchyma. Suggested modes of treatment include surgical bile duct exploration, endoscopic procedures, transhepatic cholangiolithotomy and liver resection. This paper reports 2 patients in whom liver resection was performed because of intrahepatic ductal stones, bile duct strictures and repeated episodes of cholangitis. Liver resection was uncomplicated and long-term results were satisfactory. Our results support the view that liver resection is indicated in rare instances of intrahepatic bile duct stones associated with bile duct strictures.


*HPB Surgery* 1990, Vol. 2 pp. 145–147
Reprints available directly from the publisher
Photocopying permitted by license only

© 1990 Harwood Academic Publishers GmbH
Printed in the United Kingdom

### CASE REPORT
### LIVER RESECTION FOR INTRAHEPATIC STONES

ROLAND ANDERSSON*, KARL-GÖRAN TRANBERG and STIG BENGMARK

*Department of Surgery, Lund University, S-221 85 Lund, Sweden*

*(Received 15 May 1989)*

Intrahepatic stones are difficult to manage, especially when they are associated with bile duct stricture, cholangitis and destruction of liver parenchyma. Suggested modes of treatment include surgical bile duct exploration, endoscopic procedures, transhepatic cholangiolithotomy and liver resection. This paper reports 2 patients in whom liver resection was performed because of intrahepatic ductal stones, bile duct strictures and repeated episodes of cholangitis. Liver resection was uncomplicated and long-term results were satisfactory. Our results support the view that liver resection is indicated in rare instances of intrahepatic bile duct stones associated with bile duct strictures.

KEY WORDS: Bile stones, intrahepatic, liver resection

### INTRODUCTION

Patients with intrahepatic calculi are difficult to treat especially when they have associated bile duct stricture(s), repeated episodes of cholangitis and sepsis and destruction of liver parenchyma. Procedures that have been tried in the past include proximal extended bile duct exploration[1–3], cholangiolithotomy by liver split[1,3,4] and partial hepatectomy[5–8]. New instruments and techniques like flexible choledochoscopy[9], the Fogarty balloon[3] and the Dormia basket[10] have been tried and will certainly help to improve our future handling of patients with intrahepatic stones.

Experience from hepatic resection for intrahepatic ductal stones comes mainly from Asia and is based on a limited number of patients (about a hundred)[2,6–8]. Liver resection has been reported to be the preferred method of treatment when one or more of the following conditions are met: localized involvement, intrahepatic bile duct strictures and extensive liver tissue destruction[6]. This paper reports the management and outcome of liver resection in two patients with multiple hepatic stones and multiple bile duct strictures.

### CASE REPORTS

### *Case 1*

A 43-year old man, having undergone cholecystectomy in 1958 and two operations for

* Correspondence to: Roland Andersson, M.D., Department of Surgery, Lund University, S–221 85 Lund, Sweden.

choledocholithotomy in 1978, was admitted in 1981 because of choledochal stones and pancreatitis. Endoscopic retrograde cholangiopancreatography (ERCP), as well as computed tomography (CT) and ultrasonography, visualized a dilated left hepatic duct with multiple stones, strictures and saccular dilations in the left lateral segment. The strictured part of the bile duct could not be passed with wires or catheters. Repeat ERC the following year demonstrated the same pathology and, also, a choledochal stone, which was removed with a Dormia basket after endoscopic sphincterotomy. Left lateral segmentectomy was performed the next day, removing the ductular cisterns and multiple stones. Microscopic examination revealed chronic pericholangitis. The postoperative course was uneventful except for moderate pancreatitis. A residual choledochal stone was diagnosed at control ERC 7 months postoperatively. Sphincterotomy was performed (again), but the stone could not be extracted. A new ERC 2 months later did not reveal any stones. After that, the patient has been free of symptoms from the abdomen for a follow-up period of 6 years.

*Case no. 2*

This patient had had a cholecystectomy in 1953 and had suffered from intermittent abdominal pain in his right subcostal region since 1969. In 1982, at the age of 70, he developed jaundice which led to exploratory laparotomy and choledocholithotomy. Postoperative cholangiography and ERCP revealed a large number of stones within the dorsocaudal segment of the right liver lobe, central stricturing of the corresponding segmental bile duct and prestenotic cysts. The patient was referred to us for further treatment. ERCP also demonstrated a choledochal duct stone, which was removed with a Dormia basket after sphincterotomy. Control ERCP confirmed the presence of intrahepatic stones but did not show any extrahepatic stones. As before, catheters or wires could not be forced to pass the stricture. Three weeks later, the patient underwent resection of the dorsocaudal segment of the right liver lobe with removal of 120 intrahepatic stones. Microscopic examination of the resected specimen revealed extensive inflammatory and fibrotic changes. A postoperative bilio-cutaneous fistula closed after 3 weeks. The patient has not had biliary tract symptoms during the 6 years that have passed since the liver resection despite the fact that ERCP, performed 5 months after surgery, disclosed 4–5 residual stones at the confluence of the dorsocaudal and the ventrocranial segmental ducts. We have refrained from further diagnostic and therapeutic procedures because the patient suffers from symptomatic cerebrovascular disease.

### DISCUSSION

Multiple, intrahepatic stones are more common in Asia than in Sweden or other parts of the Western world. Usually, the pathogenesis is obscure, although the high incidence of bilirubin containing stones in Asia suggests that dietary factors, and possibly worm infestations, are causative in this part of the world[6,11]. Intrahepatic stones may also form because of iatrogenic stricture of the bile ducts with stasis hemolytic disease, sclerosing cholangitis, choledochal cysts or Caroli's disease. As in our, and other non-Asian, patients, intrahepatic stones usually appear in the absence of these conditions, which suggests that most intrahepatic stones have migrated from the gallbladder.

The incidence of intrahepatic stones in cholelithiasis is quite high(5– 10%)[2,5]. Although most stones pass unnoticed, a few may cause intrahepatic obstruction by mass or by erosion with infection and stricturing. A vicious circle is then established.

Reported results for liver resection of intrahepatic stones are good both with respect to operative mortality and the incidence of residual stones and postoperative cholangitis[2,5,6,8]. The low operative mortality, like the 2% reported by Choi *et al*[6], is explained by the fact that limited resections, usually a left lateral segmentectomy, are sufficient for most patients. In addition, resection is performed in a fibrotic segment of the liver with diminished blood supply in a patient with normal clotting capacity.

It is concluded that liver resection is indicated for multiple intrahepatic stones when they are associated with irreversibly strictured, undilatable and unpassable, intrahepatic ducts.

### *References*

1. Maki, T., Sato, T., Yamaguchi, Y., Sato, T. (1964) Treatment of intrahepatic gallstones. *Arch. Surg.* **88**, 260–64.
2. Sato, T., Suzuki, N. Takahashi, W., Uematsu, I. (1980) Surgical management of intrahepatic gallstones. *Ann. Surg.* **192**, 28– 32.
3. Shore, J.M., Berci, G. (1970) Operative management of calculi in the hepatic ducts. *Am. J. Surg.* **119**, 625–631.
4. Sato, T., Matsuhior, T., Suzuki, N., Takahashi, W. (1977) Results of surgical treatment for intrahepatic gallstones. Tohoku. *J. Exp. Med.* **122**, 303.
5. Adson, M.A., Nagorney, D.M. (1982) Hepatic resection for intrahepatic ductal stones. *Arch. Surg.* **117**, 611–615.
6. Choi, T.K., Wong, J. (1986) Partial hepatectomy for intrahepatic stones. *World J. Surg.* **10**, 281–186.
7. Huang, C.-C. (1959) Partial resection of the liver in treatment of intrahepatic stones. *Chin. Med. J. Engl.* **79**, 40–45.
8. Ong, G.B. (1962) A study of recurrent pyogenic cholangitis. *Arch Surg.* **84**, 199–225.
9. Choi, S., Choi, T.K., Wong, J. (1987) Intraoperative flexible choledochoscopy for intrahepatic and extrahepatic biliary calculi. *Surgery*, **101**, 571–576.
10. Mazzariello, R. (1973) Review of 220 cases of residual biliary tract calculi treated without reoperation: An eight-year study. *Surgery*, **73**, 299–306.
11. Nagase, M., Hikasa, Y., Soloway, R.D. *et al.* (1980) Gallstones in western Japan: Factors affecting the prevalence of intrahepatic gallstones. *Gastroenterology*, **78**, 684–690.

*(Accepted by L. Blumgart 22 August 1989)*

